# Education and Public Health

**Published:** 1905-10-21

**Authors:** 


### Education and Public Health.

The favourable reception given by the International Conference at Paris to Dr. Heron's report on the arrangements for school instruction in hygiene within the United Kingdom, and the general agreement of the speakers who took part in the subsequent debate with the proposition that public health might be promoted by the systematic sowing of the seeds of sanitary knowledge in elementary schools, can hardly do other than provoke the question whether such instruction might not be afforded, in a somewhat different way, to classes who, to say the least, require it quite as much as school children, and whose ignorance is attended by far more disastrous consequences. It is impossible to ignore the fact that the law is often the best schoolmaster, and that its operation in that character is greatly needed by those children of a larger growth who, in many urban and in most rural parts of provincial England, neglect with absolute impunity the duties which they undertake as members of what is facetiously called the "sanitary authority" of some hapless borough or district. Thirty years have passed away since the late Sir John Simon reported that, of the half million deaths then registered annually in England and Wales, fully one-half would be prevented if the existing knowledge of the causes of disease, as affecting large masses of population, were even reasonably well applied throughout the country. He did not attempt to give any exact statement of the total influence which preventable diseases were then exerting against the efficiency and happiness of the population; for it was only in so far as such diseases kill that their effects could be represented by numbers. To the incalculable amount of physical pain and disablement which they occasion, and of the sorrows and anxieties, the often permanent darkening of life, the frequent destitution and pauperism, which attend upon or follow such suffering, death statistics can only bear testimony by suggestion.

When these words were written, the Local Government Board had been engaged in drawing up rules for the administration of the Public Health Act of 1872, including those which were to govern the appointments and the tenure of office of the Medical Officers of Health; and it was an open secret that the Government was in fear lest the enforcement of sanitary law would be specially unpopular among the small tradesmen who mainly swayed the municipal destinies of provincial towns, and who were, as a rule, supporters of what was called liberalism. It was therefore decided that the new rules should be so framed as to reduce the operations of the Act to a minimum; and the task of framing them was withdrawn altogether from the Medical Department of the new Local Government Board, and was committed to lawyers and engineers, who, no doubt, worked under very definite instructions. One of the results of this method of procedure was to leave the majority of the Medical Officers of Health and of the Inspectors of Nuisances absolutely at the mercy of the Local Boards which appointed them, and thus to secure their prompt removal if they attempted to render the new Act a source of protection to the inhabitants of their localities. In many districts this state of things continues, and the condition of the public health in such districts is no better than it was before the Act was passed.

But the Act of 1872 is no longer to be regarded as an experiment; and the time has surely come for giving to its provisions the educational value which sound laws properly enforced are universally admitted to possess. We question whether much will be effected by teaching the principles of health to the child of the labourer as long as the labourer's employer, in his capacity of public official, is permitted to neglect his duties with regard to those principles with complete impunity, or even with the approval of persons like-minded with himself. If the defaults of all minor sanitary authorities were liable to correction by those next above them—those of the Borough Council by the County Council, and those of the County Council by the Local Government Board—a system of adult education would be set on foot which could hardly fail, within the next few years, to exert an appreciable effect upon the physique and the longevity of the population.

